# Investigating the Causes of Substandard Concrete Strength: A Macro- and Microanalysis

**DOI:** 10.3390/ma18050953

**Published:** 2025-02-21

**Authors:** Xi Du, Youliang Chen, Lantao Xu, Aiping Shen, Bo Lu, Jie Wu, Tomas Manuel Fernandez-Steeger, Rafig Azzam

**Affiliations:** 1 Department of Civil Engineering, University of Shanghai for Science and Technology, 516 Jungong Rd, Shanghai 200093, China; duxijl@163.com (X.D.);; 2 USST Center for Instrumental Analysis, University of Shanghai for Science and Technology, 516 Jungong Rd, Shanghai 200093, China; shenaiping@usst.edu.cn; 3 School of Resources and Civil Engineering, Northeastern University, Shenyang 110819, China; 4 Department of Engineering, University of Cambridge, Cambridge CB3 0FA, UK; 5 Department of Engineering Geology, Institute of Applied Geosciences, Technical University of Berlin, Ernst-Reuter-Platz 1, 10587 Berlin, Germany; 6 Department of Engineering Geology and Hydrogeology, RWTH Aachen University, Lochnerstr. 4-20 Haus A, 52064 Aachen, Germany; azzam@lih.rwth-aachen.de

**Keywords:** concrete quality, compressive strength, aggregate gradation, mineral admixtures, microscopic characterization

## Abstract

This study investigates the root causes of substandard concrete quality in a newly constructed residential complex, addressing the critical issue of compressive strength failure in structural elements. To tackle this problem, twelve core samples were extracted from affected areas and analyzed using a combination of macro-scale techniques (high-temperature heating, acid-immersion tests) and advanced microscopic methods (SEM-EDS, XRF, XRD, FTIR, TGA). The results revealed that while material proportions generally met specifications, uneven aggregate gradation and excessive use of mineral admixtures were key factors compromising strength. Microscopic analysis further identified harmful phases and chemical corrosion products, such as sulfates, which weakened the concrete matrix. These findings underscore the necessity of stringent quality control in raw material selection, aggregate gradation, and admixture dosage. The research demonstrates that integrating macro- and microanalytical methods can significantly optimize concrete mix designs, enhance durability, and prevent premature deterioration in reinforced concrete structures. This approach has broad implications for improving construction quality and ensuring the longevity of residential and infrastructure projects.

## 1. Introduction

Concrete is a widely used construction material due to its availability, versatility, and low cost [1]. It is essential in constructing urban infrastructure, highways, ports, interchanges, airports, and hydraulic structures like dams. Concrete performance directly affects these facilities’ safety, longevity, and sustainability. Over the years, researchers have made significant progress in improving the properties of concrete, particularly in areas such as high-strength and high-performance mixes [2], concrete admixtures [3], and durability [1]. Among these, research into concrete durability is especially important. Improving durability ensures that structures can meet or exceed their expected service life under normal conditions, while reducing the amount of cement used—its main binding component. This helps to lower energy consumption and the emissions of CO_2_ and other greenhouse gases during cement production [4]. However, if concrete durability decreases, it can lead to accumulated damage, cracking, and the corrosion of the steel reinforcements, ultimately reducing the structure’s ability to bear loads. Without timely intervention, such deterioration may lead to partial or total structural failure [5].

To ensure adequate durability, the production and formulation of concrete must be carefully controlled. Typically, concrete is composed of cement, coarse aggregates, fine aggregates (sand), water, and admixtures. In the pursuit of sustainable development and performance optimization, various novel concretes have emerged, such as steel fiber-reinforced concrete [6], recycled aggregate concrete [7], and lightweight concrete incorporating EPS particles [8]. These innovations have led to a deeper understanding of how composition and mixture proportions affect mechanical and durability properties. For instance, Karthik et al. [9] demonstrated that incorporating 0.38% steel fibers and 0.12% polyethylene terephthalate (PET) fibers significantly improved mechanical performance. Similarly, Yugendar et al. [10] found that adding fly ash to steel fiber-reinforced concrete enhanced its compressive and tensile strengths, albeit with minimal impact on early-age strength. In the domain of recycled aggregate concrete, Limbachiya et al. [11] reported that using 30% recycled coarse aggregates had little effect on compressive strength; however, increasing the recycled aggregate content beyond this threshold reduced mechanical properties. Cabral et al. [12] found that the use of recycled coarse aggregates significantly impacted the compressive strength of fully recycled concrete. They also observed that both recycled coarse and fine aggregates decreased the material’s elastic modulus. Additionally, for EPS lightweight concrete, Babu et al. [13] demonstrated that adding fly ash extended the period of strength development, resulting in a 35% increase in strength at 90 days compared to 28 days. Sadrmomtazi et al. [14] showed that incorporating 10% silica fume improved EPS concrete’s compressive, splitting, and flexural strengths across various strength grades. These findings highlight the importance of optimizing mix designs and material selection to achieve the desired mechanical properties and durability.

Beyond material design, diagnostic methods for concrete quality degradation remain fragmented. Apart from the macro-level mixture design, micro-level mechanisms also play a significant role in concrete durability, such as alkali–aggregate reactions (AARs) and chemical corrosion. AARs include the alkali–silica reaction (ASR) and alkali–carbonate reaction (ACR), with ASRs being the most common in engineering practice. Theories of permeability and swelling pressure can explain the expansion and cracking caused by ASRs. Additionally, chemical corrosion in environments containing sulfates, magnesium, chloride, or carbonate ions can alter concrete’s internal pore structure and phase composition, affecting its lifespan and reliability. Advanced characterization techniques are necessary to understand these microscopic effects. For instance, mercury intrusion porosimetry (MIP) can be used to analyze the pore structure and chloride ion transport properties of concrete [15]. Yang et al. [16] studied how concrete microstructure affects chloride diffusion using MIP, finding a linear relationship between pore structure parameters and chloride diffusivity. Planel et al. [17] used X-ray diffraction (XRD) and scanning electron microscopy with energy dispersive X-ray spectroscopy (SEM-EDS) to identify sulfate corrosion products such as ettringite and gypsum, and to explore how they contribute to crack formation. Zhang et al. [18] investigated the changes in mass, expansion ratio, internal ion concentrations, strength, and microstructure in concrete exposed to different concentrations of sodium sulfate solutions, identifying three stages in the evolution of mechanical performance: enhancement, incubation, and degradation. The study also noted strong coupling effects between physical and chemical damage in sulfate-rich environments. Chen et al. [19] evaluated deterioration under sulfate and mixed-salt attacks by tracking mass loss, dynamic modulus of elasticity, and compressive strength changes, finding that certain ions in the mixed solution could inhibit or delay chloride penetration, thereby extending the time before significant deterioration occurred.

Against this backdrop, the present study addresses a real-world problem where a newly constructed residential complex, comprising six shear wall structures (including a 33-story building and 5 additional towers with a total floor area of about 180,000 m^2^), exhibited substandard concrete quality according to an official assessment. Although the main structure was completed in mid-2018, subsequent tests revealed that parts of the concrete did not meet the design and code requirements, with the underlying cause remaining elusive. To bridge the gap between isolated analytical methods, this work proposes a novel cross-scale diagnostic approach that synergizes macro-mechanical testing with multi-modal microanalytical techniques. Twelve concrete specimens were extracted from beams, slabs, and columns of the problematic building. By conducting high-temperature and acid-immersion tests, the proportions of various constituents were quantitatively evaluated. Additionally, a suite of characterization techniques—SEM-EDS, X-ray fluorescence (XRF), XRD, Fourier transform infrared spectroscopy (FTIR) and thermogravimetric analysis (TGA)—was employed to identify microstructural features, chemical composition, and potential corrosion products. Integrating macro-scale mechanical test results with detailed microanalytical data enabled us to elucidate how sulfate and other corrosive species contributed to reduced uniaxial compressive strength and compromised durability.

**Figure 1:**
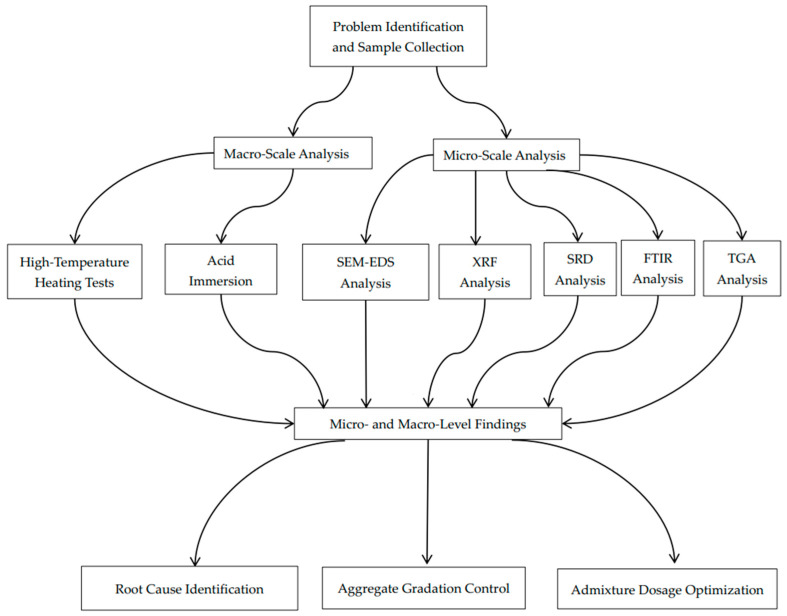
Flowchart of integrated multi-scale analysis for concrete quality degradation.

This study examines the factors leading to inadequate concrete quality in an actual engineering context. By correlating macro-level strength degradation with micro-level chemical corrosion mechanisms and combining multiple characterization methods, we offer insights into the root causes of concrete deterioration. The novelty of our approach lies in its holistic integration of macro-scale mechanical testing and microanalytical techniques to directly correlate chemical corrosion with structural degradation—an aspect often overlooked when these factors are studied in isolation. A corresponding flowchart (Figure 1) is provided to visually summarize our integrated multi-scale analysis process. These findings help explain the specific quality issues encountered in the investigated residential complex and inform future preventative measures, mixture optimization strategies, and maintenance protocols for ensuring the long-term durability and safety of reinforced concrete structures.

## 2. Specimen Preparation and Compressive Strength

### 2.1. Specimen Preparation

In this experiment, concrete samples were selected from a real engineering project with existing issues. The samples originated from the shear wall structure of a commercial residential building in a newly constructed residential area, specifically from Building 1, which comprises two underground floors and thirty-three above-ground floors. According to the conclusion of the Identification Report issued by a certain Engineering Inspection and Testing Center, the construction quality of some of the concrete did not meet the design and relevant specification requirements. However, the specific reasons for the non-conformance of the concrete could not be traced.

As shown in Table 1, 12 sample groups were selected from different locations within the residential building. The sampling method adhered to the procedures specified in the ‘Technical specification for testing concrete strength with drilled core method’ (JGJ/T384-2016) [20]. Three effective core samples were selected from each group, with each core sample having a diameter of 100 mm and a height of 100 mm. Additionally, the selected core samples underwent preliminary processing, where they were ground flat using cement mortar on a grinding machine.

### 2.2. Uniaxial Compressive Strength Test

**Figure 2:**
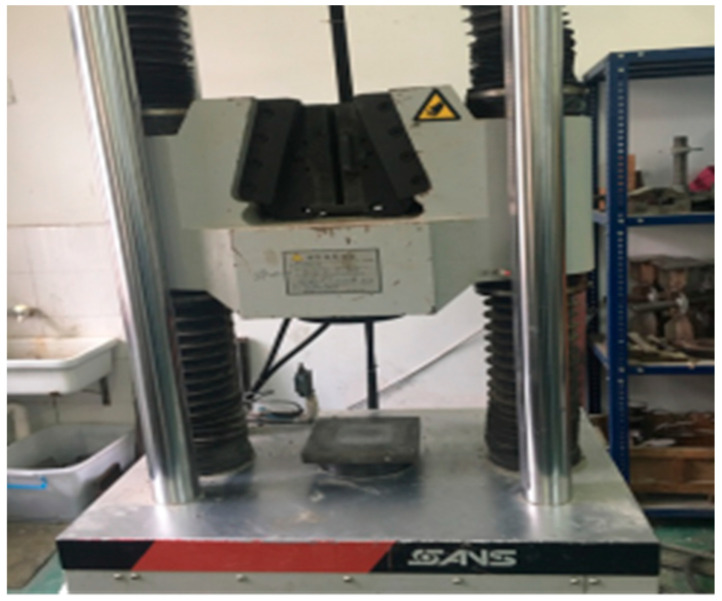
SANS microcomputer-controlled electro-hydraulic servo pressure testing machine.

The unconfined compressive strength tests were conducted using a SANS microcomputer-controlled electro-hydraulic servo pressure testing machine (Wuxi, China), as shown in Figure 2. The testing machine was computer-controlled. Initially, the upper part of the testing machine was raised using a remote-control device, and the prepared core sample was placed on the lower platen. The machine was then controlled remotely to lower the upper platen until it was close to the surface of the specimen, without making contact. Subsequently, the testing machine was started, and a constant loading rate of 50 N/s was set. Continuous loading was applied to the concrete specimen, and data were automatically collected until the specimen failed by cracking.

According to the standard mentioned above, the compressive strength test values of core samples with a height and diameter of 100 mm could be directly used as the conversion value for concrete strength. The minimum value of the concrete strength from the core sample specimens should be taken as the representative value, and as the estimated strength of the component. The experimental results are shown in Table 2.

According to the data presented in Table 2, it is observed that the compressive strength of the 12 selected concrete specimens did not meet the specified standards. To gain a deeper understanding of the factors contributing to the substandard strength of the concrete cores, this study will thoroughly analyze the compositional elements of the concrete to identify potential influencing factors.

## 3. Macroscopic Experiments on the Degradation Mechanism of Concrete

### 3.1. High-Temperature Treatment Method

In macroscopic experiments, a combination of several fundamental principles, such as elastoplastic theory, the interfacial transition zone theory, and thermodynamics, was used to determine the composition of the pre-formed concrete. The final decision was to subject the test specimens to high-temperature heating, maintaining the specimens at a specific temperature for a period to achieve the separation of the aggregates and mortar. Specific formulas were then applied to determine the proportion of each component. When the temperature exceeds 500 °C, the difference in expansion between the mortar and coarse aggregate due to the high temperature becomes significant. This expansion discrepancy causes the stress between the aggregate and mortar to reach its maximum. Specifically, the tangential shear stress reaches its peak, which primarily damages the interface between the aggregate and mortar. Additionally, at high temperatures, most of the water in the specimen evaporates. According to the interfacial transition zone theory, the bond stress reaches its minimum when the maximum shear stress forms at the interfacial transition zone. This leads to the formation of many cracks at the interface, which continue to develop under high temperatures and eventually penetrate the entire specimen. Due to these two main reasons, the strength of the aggregate and mortar in the concrete significantly decreases under high temperatures. At this temperature, the thermal compatibility between the two is the poorest, and the thermal expansion stress at their interface is the highest, leading to the maximum tangential shear stress that plays a crucial destructive role at the interface. Moreover, at this temperature, most of the combined water in ettringite and calcium hydroxide decomposes, making the interfacial transition zone structure loose. Consequently, the maximum tangential shear stress and the minimal bond stress result in new cracks forming while existing cracks expand, with some developing into penetrating cracks. The contribution of coarse aggregate to the concrete’s strength becomes minimal, and the mortar strength significantly decreases, making it easy to separate the coarse aggregate from the mortar with slight external force. Therefore, selecting 500 °C was feasible for separating coarse aggregate and mortar in hardened concrete (with carbonate as the main component of the coarse aggregate). To ensure the cracks formed at high temperatures fully expanded, the temperature was maintained for several hours, followed by the natural cooling of the specimen. Then, the mortar and coarse aggregate were manually separated.

**Figure 3:**
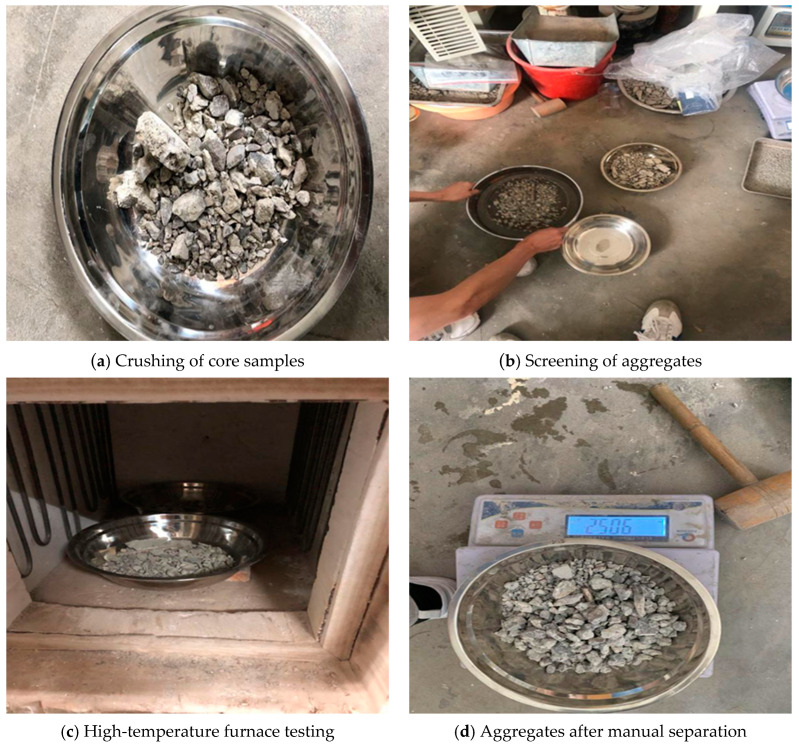
Experimental procedures for concrete aggregate testing.

Prior to testing, the core samples were crushed (Figure 3a) using a hydraulic press, controlling the crushed stone particle size to approximately 50–60 mm. The crushed stone was then placed in a high-temperature furnace and baked at 100 °C until it reached a constant weight. After removal, the crushed stone was placed in a square-hole sieve to separate out mortar blocks approximately 2.5 mm in size. These mortar blocks were placed in high-temperature-resistant metal boxes and heated in the furnace from room temperature to approximately 520 °C at a rate of about 10 °C/min. After maintaining this temperature for two hours, the heating was stopped, and the samples were cooled to room temperature. According to the experimental principles, the strength of the mortar at this point was already very low, allowing the coarse aggregate and mortar to be easily separated manually. Finally, the weights of the coarse aggregate and mortar were measured separately. The preparatory process of the experiment is shown in Figure 3.

The loss on ignition (LOI) of the core samples was calculated using Equation (1).

(1) $$\omega =\frac{m_{0}-m_{r}}{m_{0}}\times 100\%$$

where *m*_0_ is the weight of the sample before burning (in grams), *m_r_* is the weight of the sample cooled to room temperature in the desiccator after burning (in grams), and *ω* is the loss on ignition of the sample.

To minimize error, the experiment also included a correction test for the mass of the coarse aggregate. After the high-temperature heating of the crushed stone, clean coarse aggregates were selected and weighed. They were then reheated, baked to a constant weight, and weighed again to determine the LOI of the aggregates ($\omega_{1}$). The dry weight of the coarse aggregate before the 520 °C treatment for two hours was calculated using Equation (2).

(2) $$m_{c0}=\frac{m_{c}}{1-\omega_{1}}\times 100\%$$

where *m*_c0_ is the dry weight of the coarse aggregate before the 520 °C treatment for two hours (in grams), *m*_c_ is the weight of the coarse aggregate after the 520 °C treatment for two hours (in grams), and $\omega_{1}$ is the LOI of the coarse aggregate after the 520 °C treatment for two hours.

The mass of the mortar without combined water (i.e., the sum of the mass of the cement and sand) was calculated using Equation (3). The combined water content in the mortar was determined by burning the dry mortar samples at (520 ± 10) °C for three hours, then cooling them to room temperature in a desiccator. The LOI of the treated mortar after two hours at (520 ± 10) °C was calculated using Equation (1).

(3) $$m_{s1}=m_{s}\times \omega_{2}$$

where $m_{s1}$ is the mass of the mortar without combined water (in grams), $m_{s}$ is the dry weight of the separated mortar (in grams), and *ω*_2_ is the LOI of the mortar after an additional two hours at (520 ± 10) °C, totaling three hours at (520 ± 10) °C.

The relative errors of the theoretical and experimental values for the coarse aggregate and the mortar without combined water ($m_{c0}$ and $m_{s1}$) were calculated. Table 3 shows the high-temperature test results and analysis for the content of the coarse aggregate and mortar.

As shown in Table 3, the LOI of the 12 groups of core samples ranges from 2.96% to 6.96%, which is well within the acceptable limit of 8%. This indicates that the material composition ratios during mixing for the selected core samples meet the specification requirements. However, despite the compliance with the LOI standards, the observed non-compliance in concrete strength suggests that there may be an excess of undesirable substances in the mortar. While the LOI values confirm that the overall composition of the concrete samples, in terms of volatiles and water content, is consistent and controlled, factors such as the presence of impurities or an incorrect proportion of components in the mortar could weaken the overall structure. Impurities might include materials that do not contribute to the strength or durability of the concrete, such as excessive fine particles, organic matter, or unreacted cementitious materials. The high-temperature test was useful for measuring the LOI and providing a general overview of the material composition, but it did not give detailed information about the mineralogical makeup of the mortar. To accurately determine the mineral content and identify any problematic substances in the mortar, additional tests such as acid treatment and microscopic analysis were needed. These methods offer a deeper insight into the mortar’s composition, allowing us to detect any deficiencies or contaminants that could impact the strength of the concrete. Therefore, further analysis using these techniques was essential to accurately assess the mortar’s composition, identify potential issues, and gain a more complete understanding of the factors affecting concrete strength, which helps ensure that the material meets strength and durability standards.

### 3.2. Acid Solution Treatment Method

This method is based on the principle that the cement paste in concrete, which excludes the coarse and fine aggregates, will break down when exposed to a strong acid. The process began by mechanically breaking the concrete into small pieces, which were then soaked in a diluted hydrochloric acid solution to separate the aggregates. After soaking, the remaining material that was not dissolved by the acid was categorized based on particle size. Any particles larger than 4.75 mm were classified as coarse aggregates, while those between 0.15 mm and 4.75 mm were classified as fine aggregates. This allowed for the quantitative determination of the content of coarse and fine aggregates per unit volume of concrete.

Given that fly ash and other pozzolanic mineral admixtures do not participate in the hydration reaction during the early age of the concrete and are insoluble in diluted hydrochloric acid, a small sample of the cement paste could be fully dissolved in diluted hydrochloric acid. The mass of the undissolved portion could then be used to calculate the content of fly ash or other pozzolanic mineral admixtures in the concrete. According to the principle that all free water and chemically bound water in concrete materials will be lost at temperatures of 950–1000 °C, a specific amount of powdered concrete sample was subjected to high-temperature burning and weighed to obtain the total dry mass of coarse aggregates, fine aggregates, cement, and fly ash or other pozzolanic mineral admixtures in the concrete. By combining all the test data, the mass or proportion of coarse aggregate, fine aggregate, Portland cement, fly ash, or other pozzolanic mineral admixtures per unit volume of hardened concrete could be deduced, which represented the original mix proportions of the concrete.

The main steps are as follows:

(1) Calculate the volume density of concrete in an absolutely dry state.
(2) Take the concrete test samples, break them into fragments, and soak the fragments in a diluted hydrochloric acid solution to obtain slurry.
(3) Screen the mixed slurry and wash the particles on the sieve with water, collecting the slurry.
(4) Calculate the mass of fly ash in the entire test sample.
(5) Calculate the chemical-bound water content of the cement in the dry concrete.
(6) Determine the amount of coarse aggregate (G), fine aggregate (S), cement (C), and fly ash or other pozzolanic materials (FA) per cubic meter of the concrete to be tested.

The main experimental steps are as follows:

(1) Take a concrete test sample to be measured. Measure the mass of the concrete test sample in air when it is in a water-saturated surface-dry state and record this mass as *m*_1_. Then, immerse the concrete test sample in water for 24 h and measure its mass after immersion, recording this mass as *m*_2_. Next, dry the concrete test block at a temperature of 100–105 °C until it reaches a constant mass, and measure this mass as *m*_3_. Finally, calculate the absolute dry volume density of the concrete (*ρ*_0_).
  (4) $$\rho_{0}=\frac{m_{3}\rho_{w}}{m_{1}-m_{2}}$$
(2) Take another concrete test sample to be measured and crush it into fragments, sized 30–50 mm. Dry these fragments until they reach a constant mass. Weigh the dried concrete fragments and record the mass as *M*_0_. Place the *M*_0_ mass of concrete fragments into an acid-resistant plastic container and pour in a diluted hydrochloric acid solution (A) with a volume that is 2–3 times the volume of the concrete fragments. Soak for 2–3 days to obtain a slurry.
(3) Add 0.2–0.4 mol/L sodium hydroxide solution dropwise to the slurry obtained in step (2), while stirring continuously, until the pH value of the mixture reaches 6–8. Stop adding the solution and obtain the mixed slurry. Sieve the mixed slurry using a 4.75 mm square-hole sieve and wash the particles retained on the sieve 3–5 times with water. Then, sieve the slurry using a 0.15 mm square-hole sieve and wash the particles retained on this sieve 3–5 times with water. Collect all the slurry and water passing through the 0.15 mm sieve in an acid-resistant plastic container.
(4) Dry the particles retained on the 4.75 mm and 0.15 mm sieves at 100–105 °C until they reach a constant mass. Weigh these particles to obtain masses *M*_1_ and *M*_2_. Allow the solution collected in the acid-resistant plastic container in step (3) to stand and settle. Dry the sediment at 100–105 °C until it reaches a constant mass, then weigh it to obtain mass *M*_3_.
(5) Mix the powder obtained in step (4) uniformly in a ball mill, then take a powder sample with a mass of *M*_4_ (0.4–0.6 g) and place it in a beaker. Add 50 mL of distilled water and stir well, then add 40–50 mL of diluted hydrochloric acid solution (B) and continue stirring for 30–50 min. Filter the mixture using a glass sand core funnel and wash the residue on the funnel with distilled water 3–5 times. Transfer the residue to an oven and dry at 100–105 °C until it reaches a constant mass. Weigh the dried residue to obtain mass *M*_5_, and then calculate the mass of fly ash in the entire test sample (*M*_6_).
  (5) $$M_{6}=\frac{M_{5}\times M_{3}}{M_{4}}$$
(6) Dry the concrete fragments from step (2) to a constant mass. Grind the dried concrete fragments in a vibrating mill and weigh a powder sample with mass *M*_7_. Burn this powder sample in a high-temperature furnace at 950–1000 °C until it reaches a constant mass, then weigh the mass as *M*_8_. Calculate the chemical-bound water content (*β*) in the dried concrete as follows:
  (6) $$\beta =\frac{M_{7}-M_{8}}{M_{7}}\times 100\%$$
(7) Next, calculate the amounts of coarse aggregate (G), fine aggregate (S), cement (C), and fly ash or other pozzolanic materials (FA) per cubic meter of the concrete to be tested.
  (7) $$G=\frac{M_{1}}{M_{0}}\times \rho_{0}$$
  (8) $$S=\frac{M_{2}}{M_{0}}\times \rho_{0}$$
  (9) $$FA=\frac{M_{6}}{M_{0}}\times \rho_{0}$$
  (10) $$C=\left(\frac{M_{0}-M_{1}-M_{2}-M_{6}}{M_{0}}-\beta \right)\times \rho_{0}$$

The experimental results are shown in the Table 4.

In the acid solution experiments, we observed that the proportions of coarse and fine aggregates in the overall test sample were consistent with the data obtained from the high-temperature experiments. We also investigated the relationship between the coarse and fine aggregates. It was found that the strong acid in the acid solution did not react with mineral components such as fly ash in the cement mortar. This allowed us to determine the proportion of mineral admixtures, and the data indicated that the mineral admixture content exceeded the specified requirements. Additionally, during the two macro experiments, we noticed that all 12 test samples exhibited discontinuous grading of the concrete, which was one of the reasons for the failure to meet the required compressive strength.

From a broader perspective, we analyzed the components of the concrete using both high-temperature and acid-immersion experiments. The results showed that the amounts of aggregates, water, and cement mortar in the concrete were within the specified limits, indicating that the deterioration was not due to the incorrect mixing of raw materials. However, when compared with the high-temperature results, issues such as uneven grading of the coarse and fine aggregates were found, which contributed to the lower strength of the concrete. The acid-immersion experiment revealed that the amount of mineral admixtures in the concrete mortar exceeded the standard limits, which was a major cause of the concrete’s deterioration. To identify which specific minerals exceeded the limits, further microscopic analysis of core samples was necessary.

## 4. Microanalysis of Core Sample Composition

### 4.1. Scanning Electron Microscope Analysis of Concrete Samples

To analyze the composition of concrete samples, scanning electron microscopy (SEM) was used to examine various specimens. SEM is an important technique for studying the structure and morphology of inorganic materials and is commonly used in materials science [21,22].

The principle behind SEM involves scanning the sample with an electron beam. Electrons are emitted by a thermionic electron gun and, under the influence of an electric field, are accelerated and focused by multiple electromagnetic lenses to form a fine beam. This beam is directed onto the surface of the sample, and a scanning coil moves it across the surface. As the electron beam interacts with the sample, it generates various signals that provide information about the composition and structure of the material. These signals’ intensities are affected by factors such as surface features, the sample’s elemental makeup, and the strength of the electron beam. By collecting and processing these signals, detailed information about the sample can be obtained, including enhanced or selective detection of specific physical signals. Ultimately, by adjusting the electron beam’s intensity, an image reflecting the surface characteristics of the sample is displayed on the cathode ray tube (CRT) screen.

Due to the large size of the concrete samples, which exceeded the size limit for scanning electron microscopy (SEM), it was necessary to prepare specimens that met SEM requirements prior to observation. Initially, fragments smaller than 1 cm in diameter were cut from the larger concrete samples. These fragments were then mounted onto the sample holder using conductive adhesive. Subsequently, the samples underwent a carbon-coating treatment to ensure their surface conductivity. Finally, the prepared specimens were placed into the SEM chamber for observation.

**Figure 4:**
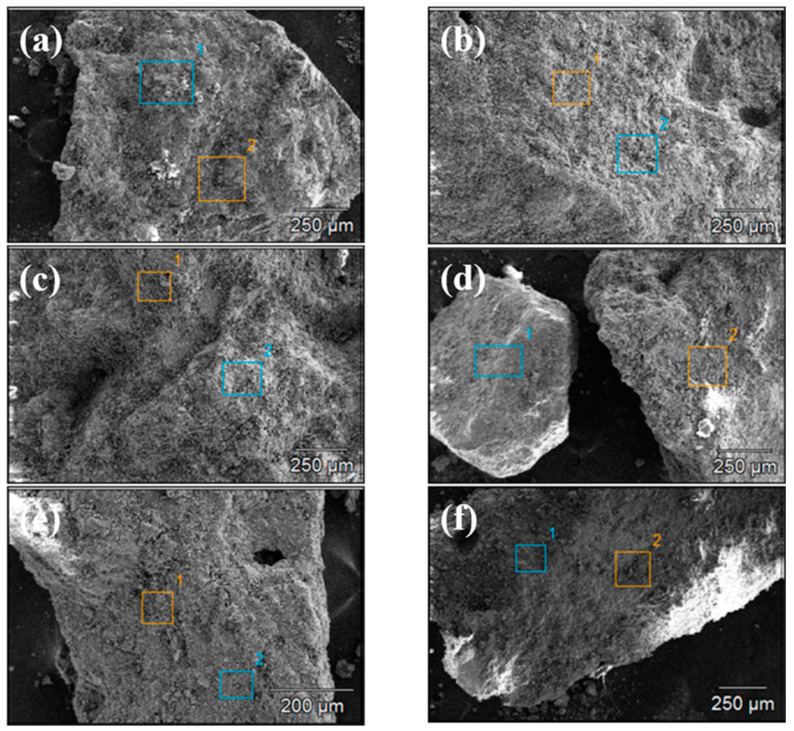
Scanning electron microscope images of concrete samples: (**a**) 1, (**b**) 2, (**c**) 3, (**d**) 4, (**e**) 5, and (**f**) 6.

**Figure 5:**
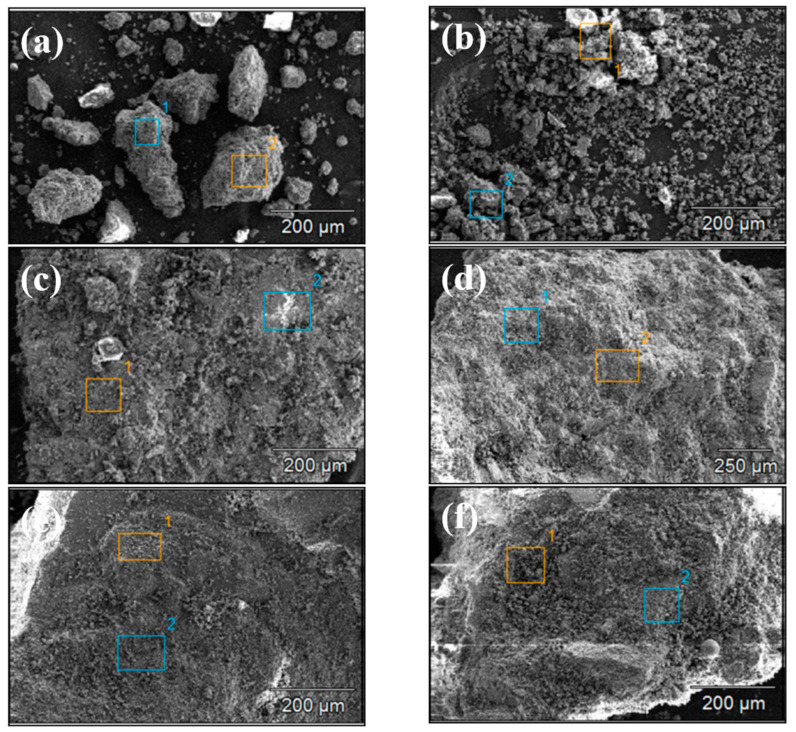
Scanning electron microscope images of concrete samples: (**a**) 7, (**b**) 8, (**c**) 9, (**d**) 10, (**e**) 11, (**f**) 12.

Figure 4 and Figure 5 show the SEM images of concrete Samples 1–12. Since these SEM images were captured in the secondary electron mode, the contrast observed in the images is independent of the sample composition. Comparing the SEM images of Samples 1–12, it is evident that Samples 7 and 8 exhibit the loosest structure, with the image showing distinct, finely fragmented particles, in contrast to the other samples. With the exception of Samples 7 and 8, the SEM specimens of the other samples predominantly consist of larger chunks, within which numerous fine particles can be observed.

### 4.2. Energy Dispersive X-Ray Spectroscopy of Concrete Samples

During the observation of the samples, energy dispersive X-ray spectroscopy (EDX), an integrated feature of the scanning electron microscope (SEM), was also employed to analyze the elemental composition of the concrete samples.

The ability of EDX to analyze the elemental composition of materials is based on the principle that, when the sample is bombarded with a high-energy electron beam under accelerated voltage, electrons from the inner shells of the atoms are ejected, creating vacancies. This results in an unstable excited state within the atom. Consequently, outer-shell electrons undergo transitions to fill the inner vacancies, releasing energy in the process. This energy, which is quantized, manifests as characteristic X-ray radiation. A portion of the energy excites outer-shell electrons, resulting in the emission of Auger electrons. The minimum energy required to excite electrons from their respective shells is known as the critical excitation energy. Since the binding energies between the nucleus and the electrons in different shells are fixed, the critical excitation energy is also a fixed value. The energy of the characteristic X-rays produced corresponds to the difference between the critical excitation energies of the involved shells. For instance, if an electron from the L-shell transitions to fill a vacancy in the K-shell, the resulting X-ray energy is the difference between the critical excitation energies of the K- and L-shells. The energy of these X-rays is unique to each element, reflecting their characteristic natures. Therefore, there is a functional relationship between the energy of the characteristic X-ray (E) and the atomic number (Z) of the constituent element. By detecting the energy of a specific characteristic X-ray, the atomic number corresponding to that energy can be identified, thereby revealing the element being analyzed. This is the theoretical basis for elemental analysis using characteristic X-ray emissions.

**Figure 6:**
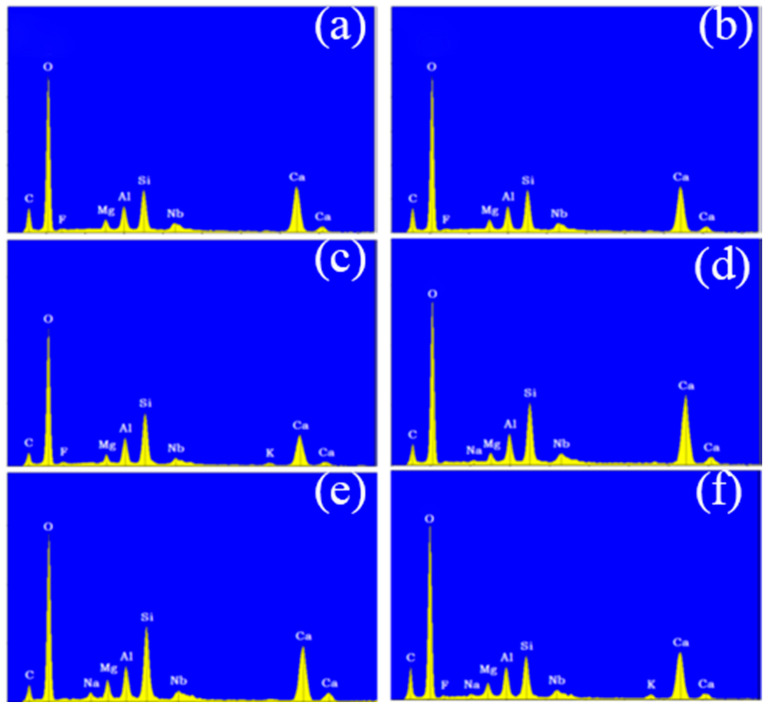
Energy dispersive X-ray spectroscopy of concrete samples: (**a**) 1, (**b**) 2, (**c**) 3, (**d**) 4, (**e**) 5, and (**f**) 6.

**Figure 7:**
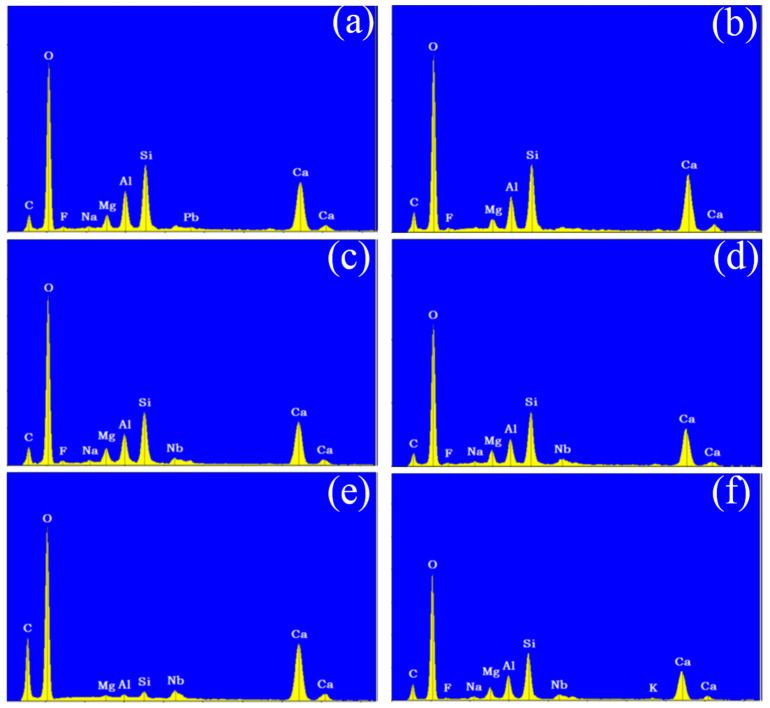
Energy dispersive X-ray spectroscopy of concrete samples: (**a**) 7, (**b**) 8, (**c**) 9, (**d**) 10, (**e**) 11, and (**f**) 12.

Following the surface morphology analysis, EDX spectrum analysis was conducted on two distinct regions of each concrete sample, represented by the blue and orange areas in the images. The results are shown in Figure 6 and Figure 7. A comparison of the EDX spectra of concrete Samples 1 to 12 reveals that the spectra are similar, indicating that the compositions of the different samples are comparable. As an example, the EDX spectrum of Sample 1 was selected for a detailed elemental composition analysis.

The EDX spectrum of Sample 1 displays nine characteristic peaks, which, when compared with standard elemental peaks, correspond to O, Ca, Si, C, Al, Mg, Nb, and F elements. Therefore, it can be concluded that concrete Samples 1 to 12 contained O, Ca, Si, C, Al, Mg, and Nb elements, with their concentrations decreasing in the order listed. Although a characteristic peak for F was observed in the EDX spectrum, its low intensity suggests that F was either absent or present in trace amounts in the concrete samples.

### 4.3. X-Ray Fluorescence Spectroscopy of Concrete Samples

After obtaining the elemental composition of the concrete samples, X-ray fluorescence (XRF) spectroscopy was employed for further quantitative analysis of the elements in the concrete samples.

X-ray fluorescence spectroscopy is capable of analyzing the elemental content of materials due to the ability of X-rays to excite atoms. The energy of an electron within an atom is determined by the element to which it belongs and its position within the electron shell. When atoms are irradiated with high-energy X-ray photons or electrons of sufficient intensity, electrons are ejected from their original positions within the atom, creating vacancies in the inner shells, such as the K-shell. This vacancy places the atom in an unstable high-energy state. To regain stability, outer-shell electrons must transition to fill the vacancy in the inner shell. For example, when an electron from the L-shell transitions to the K-shell, excess energy is released in the form of an X-ray photon. This emission appears as a line in the fluorescence spectrum. The energy difference between the vacancy in the shell and the L- and K-shells determines the intensity of the X-ray fluorescence. Through the characteristic X-ray fluorescence of various elements in the periodic table, a specific relationship between X-ray fluorescence intensity and atomic number is established. Extensive experiments have demonstrated that the characteristic X-ray fluorescence of elements from aluminum to gold is proportional to their atomic number. Thus, by analyzing the X-ray fluorescence spectrum with the appropriate algorithms and using calibration curves, the elements present in the samples could be determined [23,24].

Unlike scanning electron microscopy, X-ray fluorescence testing has no strict requirements for the sample, other than the need for a relatively smooth surface. Therefore, for the X-ray fluorescence analysis, the concrete samples were simply polished using 2000-grit sandpaper. After polishing to obtain a smooth surface approximately 1 cm × 1 cm in size, analysis was conducted. The X-ray fluorescence spectrometer used for testing was the Thermo Scientific ‘ARL AdvantX Intellipower™ 4200’ model (Waltham, MA, USA).

The X-ray fluorescence results for the concrete samples are shown in Table 5.

From Table 5, it can be observed that the primary elements in the concrete samples are Ca, Si, Al, Fe, Mg, and Na. The elemental compositions of concrete Samples 1 to 12 were as follows:

For Sample 1, the contents of Ca, Si, Al, Fe, Mg, Na, S, K, and Ti were 35.59%, 13.10%, 4.57%, 3.60%, 3.16%, 3.60%, 0.325%, 0.521%, and 0.383%, respectively.

For Sample 2, the contents are 35.48%, 12.12%, 5.71%, 3.43%, 2.16%, 1.05%, 0.561%, 0.990%, and 0.325%, respectively.

For Sample 3, the contents were 31.93%, 13.32%, 6.71%, 3.88%, 2.30%, 0.463%, 0.541%, 1.40%, and 0.409%, respectively.

For Sample 4, the contents were 35.66%, 13.91%, 4.81%, 2.98%, 1.52%, 0.891%, 0.414%, 0.818%, and 0.313%, respectively.

For Sample 5, the contents were 34.71%, 11.81%, 5.78%, 2.59%, 3.05%, 1.58%, 0.531%, 1.65%, and 0.385%, respectively.

For Sample 6, the contents were 34.31%, 13.20%, 6.12%, 2.85%, 2.41%, 0.558%, 0.381%, 1.17%, and 0.334%, respectively.

For Sample 7, the contents were 33.82%, 11.83%, 6.51%, 3.90%, 2.48%, 1.08%, 0.513%, 1.14%, and 0.440%, respectively.

For Sample 8, the contents were 36.66%, 10.96%, 5.62%, 3.63%, 2.07%, 1.51%, 0.380%, 1.69%, and 0.410%, respectively.

For Sample 9, the contents were 36.33%, 10.47%, 5.73%, 4.29%, 3.12%, 0.729%, 0.479%, 1.10%, and 0.431%, respectively.

For Sample 10, the contents were 36.74%, 10.29%, 5.38%, 4.10%, 3.36%, 0.860%, 0.430%, 1.36%, and 0.469%, respectively.

For Sample 11, the contents were 34.90%, 12.69%, 3.98%, 4.98%, 1.75%, 0.471%, 0.530%, 2.67%, and 0.401%, respectively.

For Sample 12, the contents were 30.66%, 12.51%, 6.92%, 4.58%, 3.54%, 0.566%, 0.549%, 1.11%, and 0.509%, respectively.

These results are similar to those obtained from the energy dispersive X-ray spectroscopy (EDX) analysis.

The element oxygen (O) is absent from the X-ray fluorescence spectrometry results for the concrete samples, which is due to the fact that EDX analysis is not capable of detecting elements with low atomic numbers.

**Figure 8:**
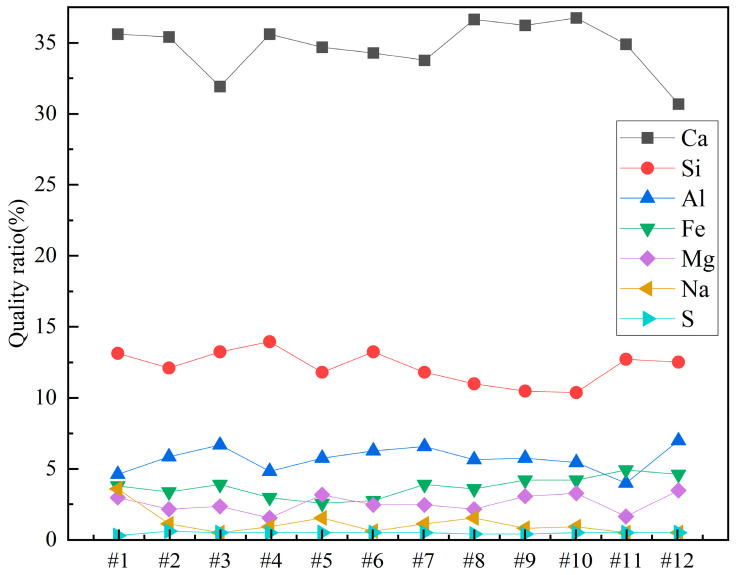
Weight percentages of elements in concrete samples.

Since it is not immediately clear from the table, the contents of Ca, Si, Al, Fe, Mg, Na, and S in the different concrete samples were plotted for clarity. The results are shown in Figure 8.

From Figure 8, it can be observed that among all the concrete samples, Ca content is the highest, accounting for more than 30%. Sample 12 has the lowest Ca content, approximately 30%, while Samples 8 and 10 have the highest Ca contents, around 35%. Si content is about 12% in all the concrete samples. It can also be noted that Si content slightly decreases as the sample number increases from 1 to 12. Al content is around 5% in all the samples, with the lowest Al content in Sample 1 and the highest in Sample 12. The contents of Fe, Mg, and Na are all less than 5%. Among Samples 1 to 12, Fe content shows a slight increase, while Na content shows a slight decrease as the sample number increases. The S element plays an important role in the properties of concrete, and its content in the samples is approximately 0.5%. Sample 1 has the lowest S content, at 0.325%, while Sample 2 has the highest S content, at 0.561%.

### 4.4. FTIR Analysis of Concrete Samples

**Figure 9:**
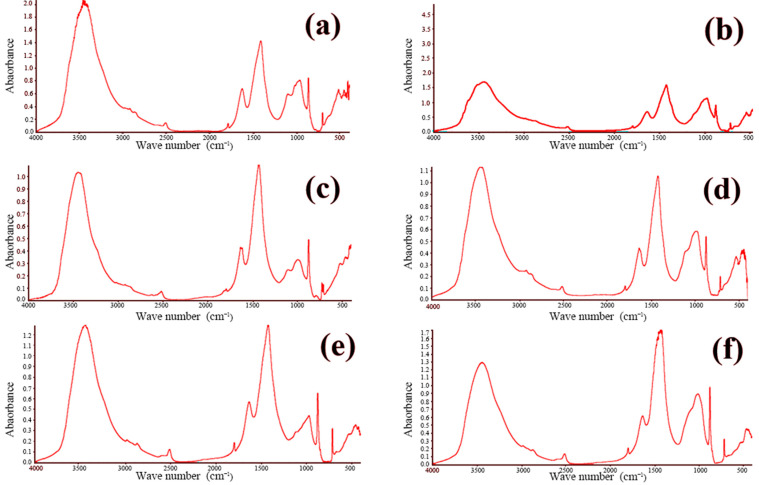
FTIR infrared spectra of concrete samples: (**a**) 1, (**b**) 2, (**c**) 3, (**d**) 4, (**e**) 5, and (**f**) 6.

**Figure 10:**
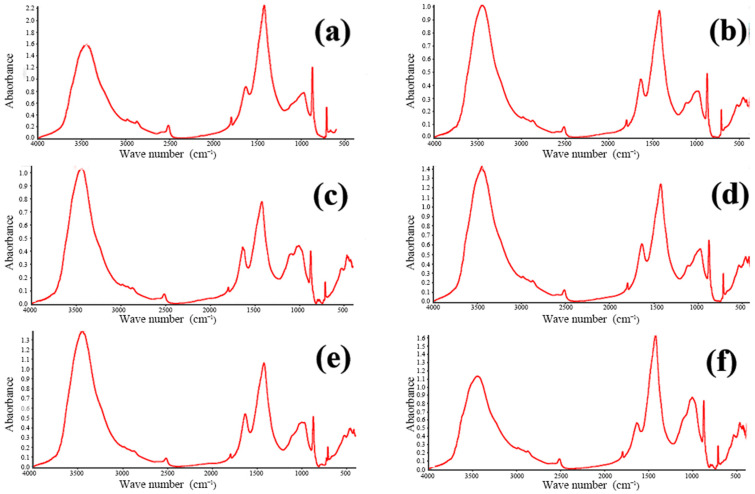
FTIR infrared spectra of concrete samples: (**a**) 7, (**b**) 8, (**c**) 9, (**d**) 10, (**e**) 11, and (**f**) 12.

FTIR is widely employed for both the qualitative and quantitative assessment of functional groups in organic and inorganic materials. The technique operates by passing an infrared beam from the source through a Michelson interferometer, where the beam undergoes interference, and subsequently directing the modulated beam to a detector. After the Fourier transform processing of the recorded interferogram, the characteristic infrared absorption peaks of the sample are obtained. By examining these absorption features, one can identify the functional groups present within the material. The concrete samples were analyzed using a Nicolet iS-5 FTIR spectrometer (Waltham, MA, USA). The resulting spectra are presented in Figure 9 and Figure 10. These figures showcase the FTIR spectra of all twelve concrete samples, revealing similar characteristic absorption peaks with no appreciable differences in peak intensities. This uniformity strongly suggests that the phase compositions of the samples are essentially the same, which is consistent with the previously obtained XRD data.

**Figure 11:**
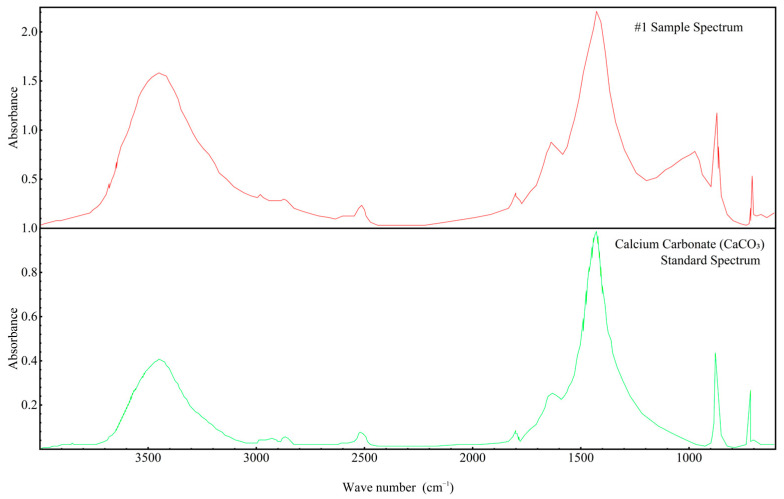
FTIR infrared spectrum of concrete Sample 1 and the standard spectrum of calcium carbonate (CaCO_3_).

Since the FTIR spectra of all samples were found to be similar, the spectrum of Sample 1 was selected for more detailed analysis, as shown in Figure 11. This figure compares the FTIR spectrum of Sample 1 with the standard spectrum of calcium carbonate (CaCO_3_). The key absorption bands of Sample 1 correspond closely to those of CaCO_3_, particularly in the regions around 1400–1500 cm^−1^ (C–O stretching) and 850–900 cm^−1^ (C–O bending). These matching absorption features confirm the presence of carbonate ions (CO_3_^2−^), consistent with the molecular structure of CaCO_3_.

By comparing the upper spectrum of Sample 1 to the lower CaCO_3_ standard spectrum, it can be conclusively demonstrated that calcium carbonate is indeed a component of the concrete samples. This observation aligns with the earlier XRD and X-ray fluorescence (XRF) analyses and provides functional group-level confirmation of the sample’s phase composition. Moreover, the similarity in overall peak profiles and positions between the sample and the standard indicates that CaCO_3_ is a dominant species within the concrete. These results further reinforce the previous inferences regarding the origin and binding state of calcium and carbon in the tested samples.

**Figure 12:**
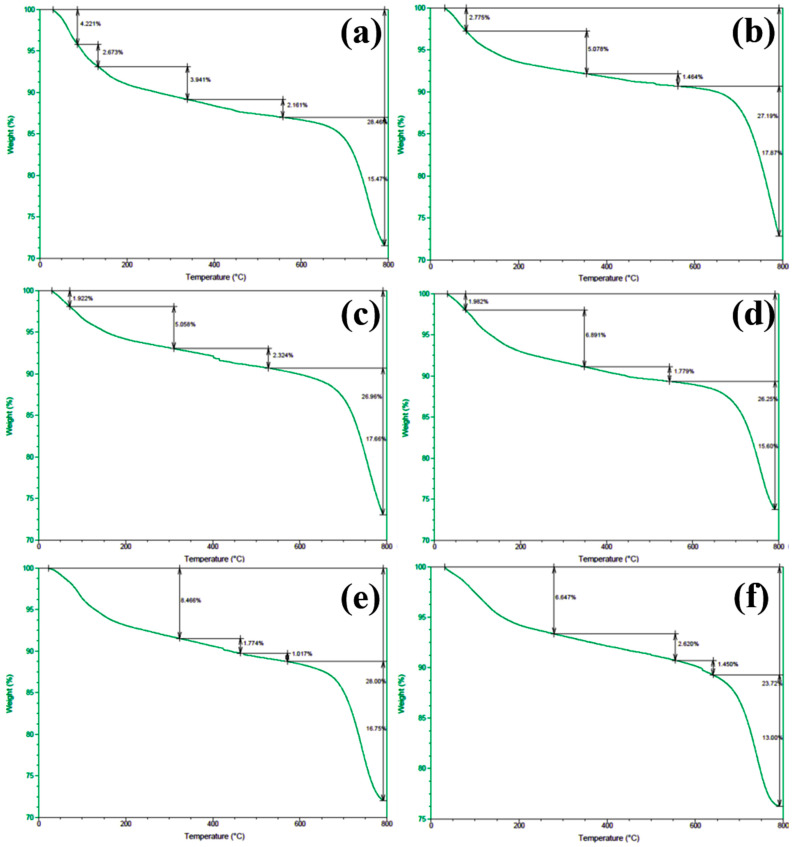
TGA spectra of concrete samples: (**a**) 1, (**b**) 2, (**c**) 3, (**d**) 4, (**e**) 5, and (**f**) 6.

**Figure 13:**
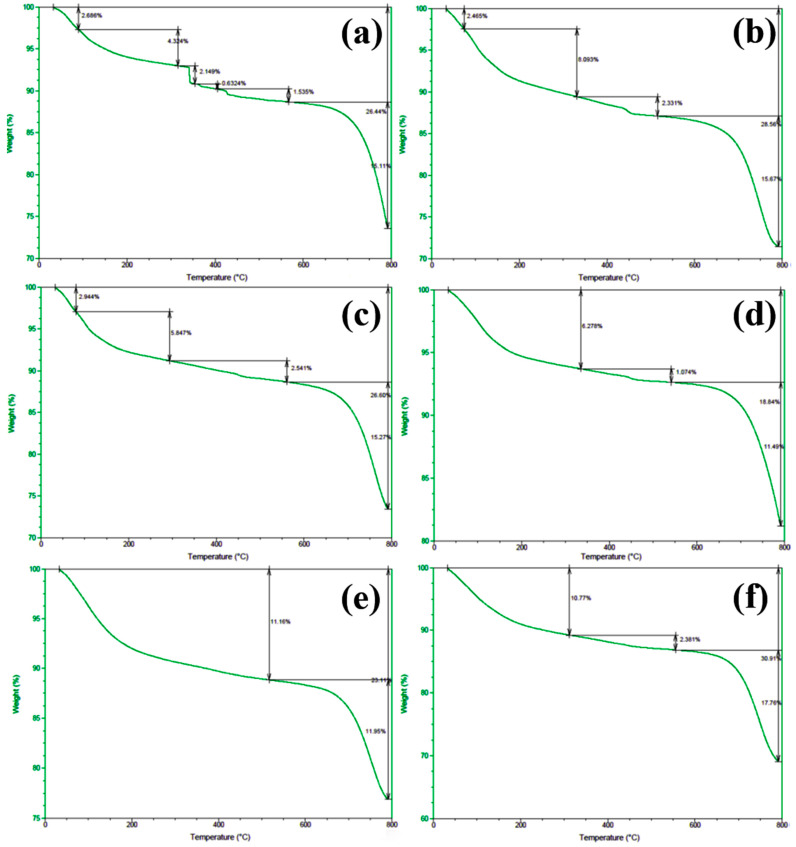
TGA spectra of concrete samples: (**a**) 7, (**b**) 8, (**c**) 9, (**d**) 10, (**e**) 11, and (**f**) 12.

**Figure 14:**
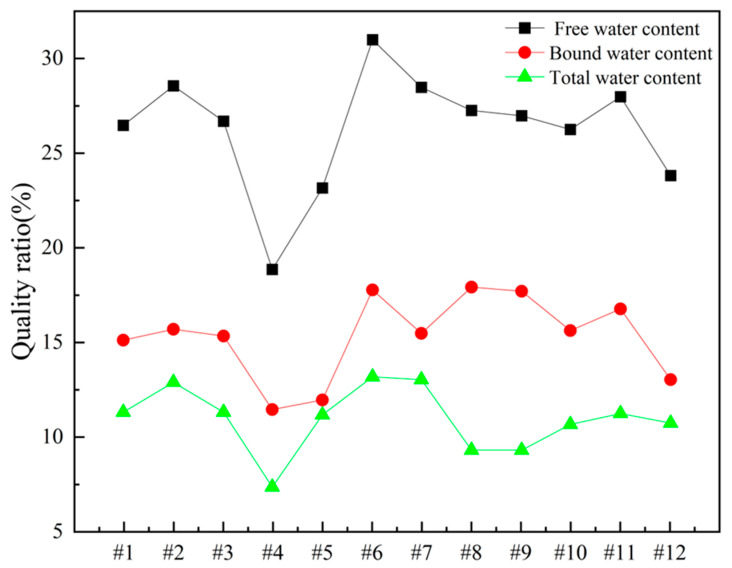
Water content of concrete samples.

In order to gain deeper insights into the decomposition behavior of the concrete samples during heating and to elucidate the distribution of water phases (free water and bound water), thermogravimetric analysis (TGA) was performed on the series of differently numbered concrete specimens. The measurements were taken using a TA-Q500 TGA instrument, with the temperature ranging from ambient conditions (approximately 25 °C) to 800 °C at a heating rate of 20 °C/min. The resulting TGA curves are presented in Figure 12 and Figure 13, where Figure 12 displays the curves for Samples 1 through 6, and Figure 13 shows those for Samples 7 through 12. To clearly illustrate the various forms and contents of water present in each sample, the free and bound water contents derived from the TGA data were further quantified, and the results are summarized in Figure 14.

An examination of the TGA curves reveals that substantial mass loss occurs in the initial stages of heating. In particular, a rapid weight reduction is observed before the temperature reaches approximately 200 °C. This low-temperature weight loss is generally attributed to the evaporation of free water, which is only weakly held in the pore structure or adsorbed onto solid surfaces. As the temperature rises to the 100–200 °C range, these loosely bound water molecules readily escape from the material, resulting in a noticeable decrease in sample mass.

As heating continues, another pronounced weight loss occurs around 600 °C, indicating a more intense decomposition process at higher temperatures. This high-temperature mass loss is commonly associated with the release of bound water, which is more firmly incorporated into mineral lattices or the structure of hydrated phases. The liberation of these water molecules requires substantially higher energy, and thus, significant mass loss is only observed at elevated temperatures beyond approximately 600 °C.

To quantitatively assess the distribution of water phases in the concrete samples, the TGA data were further processed to determine the relative contents of free water and bound water in each specimen. The results of this analysis are presented in Figure 14, which provides a clear and direct visualization of the water distribution and proportions across the samples. This information offers valuable insights into understanding how the water content correlates with critical material properties such as porosity, density, and durability, which are known to have significant impacts on the overall performance and longevity of concrete structures. The TGA curves presented in Figure 12 and Figure 13, in conjunction with the water content distribution shown in Figure 14, collectively substantiate the distinct release patterns and kinetic characteristics of free and bound water during thermal decomposition. These findings offer valuable insights into the thermal stability and microstructural evolution of the concrete under elevated-temperature conditions.

## 5. Conclusions and Discussion

In this study, a comprehensive multi-scale investigation was conducted to identify the underlying causes of substandard compressive strength in concrete extracted from an actual engineering project. The following key points summarize the main findings and contributions of this work:

(1) Utilizing high-temperature and acid-immersion tests, we confirmed that while the overall mix proportion of the concrete samples nominally met standard specifications, discontinuous aggregate gradation and excessive mineral admixture content contributed to an unexpected reduction in uniaxial compressive strength.
(2) Analytical techniques—SEM-EDS, XRF, XRD, FTIR, Py-GC/MS, and TGA—revealed that despite general compositional compliance, the presence of corrosive species (e.g., sulfates) and non-ideal phases in the cementitious matrix undermined the concrete’s internal structure. These factors, often undetectable by routine inspections, were shown to weaken the interfacial transition zones and promote microcracking and pore connectivity.
(3) This study highlights that mere adherence to standard mix proportions is insufficient for long-term durability, as environmental corrosion, suboptimal mineral admixture content, and uneven aggregate distribution degrade structural integrity. Consequently, a more holistic quality assurance approach is required—one integrating microstructural analysis, environmental assessments, and strict admixture controls—to prevent premature deterioration and ensure structures attain their designed service life.

However, there are several limitations in this study. The sample selection was limited to a single engineering project, and thus, the results may not be fully representative of concrete from other regions or under different environmental conditions. Further studies are needed to validate the findings across a wider range of concrete mixtures and applications. The investigation focused on a limited set of analytical techniques. While powerful, these methods may not capture all potential contributors to concrete degradation, such as the effects of localized microenvironments or aging-related changes. Incorporating additional non-destructive testing methods could provide a more complete picture of long-term concrete performance. The study did not address the economic feasibility of implementing more refined mix designs and diagnostic approaches on a larger scale. The cost-effectiveness of integrating more advanced diagnostic tools and adjusting mix designs in routine construction practices should be explored in future research.

These findings indicate that simply relying on standard mix design parameters does not guarantee optimal concrete performance. Although preliminary tests confirmed that basic component ratios and water content met the required specifications, discrepancies between macro-level metrics and in-service strength highlight subtle yet often overlooked microstructural and chemical factors.

Mineral admixtures, commonly added to improve workability, durability, and cost-effectiveness, may become counterproductive if their type or dosage is not well aligned with the aggregate system and service environment. Excessive or improperly selected admixtures alter the composition of hydration products, weaken the interfacial transition zones, and facilitate the ingress of corrosive ions. When combined with discontinuous aggregate grading, these chemical and physical imbalances concentrate internal stresses, promote microcracking, and create a self-reinforcing cycle of deterioration.

The presence of sulfates and other corrosive products further underscores the limitations of traditional quality control methods based solely on standard mix proportions and conventional curing conditions. In complex service environments, such design margins may be insufficient. Adopting multi-scale diagnostic approaches—such as non-destructive microanalysis, periodic inspections, and data-driven predictive models—enables proactive assessment and intervention against deterioration.

Additionally, premature structural failure significantly increases the environmental and economic costs of repair, retrofitting, or reconstruction. Integrating microstructural considerations and environmental simulations at the design stage can reduce the risk of early failure, lower maintenance demands, and enhance the sustainability of concrete infrastructure.

Overall, this study emphasizes the importance of a multi-level, holistic analytical approach. It offers valuable insights for improving current practices, encouraging the use of more refined analytical techniques to ensure concrete quality and long-term durability.

## Figures and Tables

**Table 1:** Sources of 12 experimental samples.

Specimen Number	Floor	Component	Core Sample Diameter (mm)	Core Sample Height (mm)
1	25F	Slab	100	100
2	13F	Column	100	100
3	9F	Column	100	100
4	12F	Shear Wall	100	100
5	6F	Shear Wall	100	100
6	3F	Shear Wall	100	100
7	29F	Shear Wall	100	100
8	20F	Shear Wall	100	100
9	33F	Shear Wall	100	100
10	29F	Shear Wall	100	100
11	26F	Beam	100	100
12	25F	Roof Slab	100	100

**Table 2:** Uniaxial compressive strength of core samples.

No.	Compressive Strength (MPa)			Measurement Uncertainty (MPa) (at 95% Confidence (*k* = 2))	Estimated Strength (MPa)	Certificate Original Strength (MPa)	Design Strength
1	25.61	27.94	31.91	1.84	25.61	36.60	C30
2	21.67	22.11	23.89	0.68	21.67	50.10	C40
3	23.46	27.01	24.92	1.03	23.46	50.50	C40
4	23.31	22.96	21.36	0.60	21.36	50.50	C40
5	40.25	34.35	39.61	1.87	34.35	50.50	C40
6	34.69	37.55	52.71	5.59	34.69	55.60	C45
7	19.99	19.64	20.14	0.15	19.64	37.00	C30
8	25.92	21.60	23.85	1.25	21.60	43.70	C35
9	24.80	26.91	25.24	0.64	24.80	37.60	C30
10	20.14	25.02	19.57	1.73	19.57	37.60	C30
11	19.35	24.22	18.27	1.83	18.27	36.80	C30
12	19.15	14.27	18.16	1.49	14.27	37.10	C30

**Table 3:** Results and analysis of high-temperature tests for coarse aggregate and mortar content.

Core Sample No.	Weight Before Burning *m*_0_ (g)	Weight After Burning *m_r_* (g)	Coarse Aggregate Weight *m*_c_ (g)	Mortar Weight *m*_s_ (g)	LOI *ω*_1_
1	532.0	515.4	435.3	80.1	3.12%
2	327.4	312.6	236.2	76.4	4.52%
3	359.5	344.2	298.9	45.3	4.26%
4	511.6	493.2	410.2	83.0	3.60%
5	544.7	522.2	444.7	77.5	4.13%
6	1035.7	979.5	795.6	183.9	5.43%
7	308.9	298.2	228.4	69.8	3.46%
8	252.7	238.9	167.7	71.2	5.46%
9	504.6	485.3	370.9	114.4	3.82%
10	378.8	367.6	275.2	92.4	2.96%
11	380.1	368.8	316.1	52.7	2.97%
12	929.4	864.7	751.7	113.0	6.96%

**Table 4:** Results and analysis of cement and mineral admixture content by acid solution treatment.

Sample No.	Β (%)	G (g)	S (g)	C (g)	FA (g)	Mineral Admixture Proportion (%)
1	30.26	52.83	9.85	7.10	0.45	6.33
2	33.33	85.28	26.24	9.87	0.51	5.17
3	21.92	120.80	20.08	11.84	0.88	7.43
4	35.26	133.19	35.17	17.03	1.22	7.16
5	34.92	61.52	19.05	10.52	0.82	7.79
6	27.17	73.80	16.57	7.00	0.71	10.14
7	26.42	56.14	6.73	6.21	0.61	9.82
8	19.08	39.35	1.81	4.67	0.56	11.99
9	23.78	48.02	5.83	4.73	0.53	11.20
10	29.45	48.74	4.12	6.82	0.45	6.60
11	25.67	64.78	11.02	6.59	0.48	7.28
12	27.55	45.98	8.69	4.51	0.42	9.31

**Table 5:** X-ray fluorescence spectrometer test results for concrete samples (wt.%).

	1	2	3	4	5	6	7	8	9	10	11	12
Ca	35.59	35.48	31.93	35.66	34.71	34.31	33.82	36.66	36.33	36.74	34.90	30.66
Si	13.10	12.12	13.32	13.91	11.81	13.20	11.83	10.96	10.47	10.29	12.69	12.51
Al	4.57	5.71	6.71	4.81	5.78	6.12	6.51	5.62	5.73	5.38	3.98	6.92
Fe	3.60	3.43	3.88	2.98	2.59	2.85	3.90	3.63	4.29	4.10	4.98	4.58
Mg	3.16	2.16	2.30	1.52	3.05	2.41	2.48	2.07	3.12	3.36	1.75	3.54
Na	3.60	1.05	0.463	0.891	1.58	0.558	1.08	1.51	0.729	0.860	0.471	0.566
S	0.325	0.561	0.541	0.414	0.531	0.381	0.513	0.380	0.479	0.430	0.530	0.549
K	0.521	0.990	1.40	0.818	1.65	1.17	1.14	1.69	1.10	1.36	2.67	1.11
Ti	0.383	0.325	0.409	0.313	0.385	0.334	0.440	0.410	0.431	0.469	0.401	0.509

## Data Availability

The original contributions presented in this study are included in the article. Further inquiries can be directed to the corresponding author.
